# Luteal Phase Support in the Intrauterine Insemination (IUI) Cycles: A Randomized Double Blind, Placebo Controlled Study

**Published:** 2014-12

**Authors:** Batool Hossein Rashidi, Fatemeh Davari Tanha, Haleh Rahmanpour, Mahya Ghazizadeh

**Affiliations:** 1 Reproductive Health Research Center, Tehran University of Medical Sciences, Tehran, Iran; 2 Zanan Hospital, Tehran University of Medical Sciences, Tehran, Iran; 3 Zanjan University of Medical Sciences, Zanjan, Iran

**Keywords:** clomiphene citrate, hMG, IUI, luteal phase support, progesterone

## Abstract

**Objective:** To evaluate the impact of luteal phase support with vaginal progesterone on pregnancy rates in the intrauterine insemination (IUI) cycles, stimulated with clomiphene citrate and human menopausal gonadotropin (hMG), in sub fertile couples.

**Materials and methods:** This prospective, randomized, double blind study was performed in a tertiary infertility center from March 2011 to January 2012. It consisted of 253 sub fertile couples undergoing ovarian stimulation for IUI cycles. They underwent ovarian stimulation with clomiphene citrate (100 mg) and hMG (75 IU) in preparation for the IUI cycle. Study group (n = 127) received luteal phase support in the form of vaginal progesterone (400 mg twice a day), and control group (n = 126) received placebo. Clinical pregnancy and abortion rates were assessed and compared between the two groups.

**Results:** The clinical pregnancy rate was not significantly higher for supported cycles than that for the unsupported ones (15.75% vs. 12.69%, p = 0.3). The abortion rate in the patients with progesterone luteal support compared to placebo group was not statistically different (10% vs. 18.75%, p = 0.45).

**Conclusion:** It seems that luteal phase support with vaginal progesterone was not enhanced the success of IUI cycles outcomes, when clomiphene citrate and hMG were used for ovulation stimulation.

## Introduction

Intrauterine insemination (IUI) is amongst the most recommended procedures to enhance the probability of pregnancy in couples with sub fertility. The success rate of this technique depends on numerous factors, one of which is the quality of the luteal phase (1). Luteal phase deficiency (LPD) may be associated with insufficient production of progesterone, which is essential for embryo implantation and maintenance of early pregnancy (2). 

Clinical conditions such as stress, polycystic ovary syndrome (PCOS), aging, ovulation stimulation, ovulation induction with or without gonadotropin releasing hormone (GnRH) agonists, and assistant reproductive technologies (ART) may manifest as a LPD status (3-9). 

Controlled ovarian hyper stimulation also results in multifollicular development with higher steroid serum concentrations, compared with natural cycles. It is assumed that supraphysiologic serum steroid concentrations might adversely affect LH secretion via feedback mechanisms, which in turn results in premature luteolysis and defective progesterone secretion (10).

Support of the luteal phase with vaginal or intramuscular progesterone is the only recognized treatment in order to escalate the clinical outcomes of stimulated IVF/intracytoplasmic sperm injection (ICSI) cycles. However, questions about the necessity of luteal support in stimulated IUI cycles remain unanswered (11-14). Unlike IVF/ICSI cycles, GnRH agonist is not administered, follicles are not aspirated, and only two or three corpus luteum are produced after ovulation stimulation in the IUI cycles. Hence, luteal phase support may not be necessary in IUI cycles (15).

In a systematic review and meta-analysis by Hill MJ et.al in 2013, they concluded that luteal phase support may be of benefit to patients undergoing ovulation induction with gonadotropins but not clomiphene citrate in IUI cycles (16).

The aim of this study was to evaluate the impact of luteal phase support on IUI cycle outcomes stimulated with clomiphene plus hMG in sub fertile couples.

## Materials and methods

This prospective, randomized, double blind study was performed in a tertiary infertility center from January 2012 to December 2012. It consisted of 253 sub fertile couples undergoing ovarian stimulation for IUI cycles. Inclusion criteria were: age 20-35 years, normal hormonal assay, normal pelvis in transvaginal sonography, duration of infertility ≤ 5 years, and bilateral tubal patency at hystrosalpingography. Exclusion criteria were: Basal levels of FSH≥10mlU/ml, endometriosis stage 3, 4, or a history of pelvic surgery and severe male factor infertility. The patients were randomly divided into A or B groups based on a computer generated list, while neither the patients nor the procedure developer had any information about the treatment assignment. Before the project commenced, the study was approved by the University Ethics Committee.

In all patients a transvaginal ultrasonography (TVS) was performed on the third day of their menstrual cycle, and 100 mg of clomiphene citrate oral tablet (Iran Hormone Co., Tehran, Iran) was administered if both ovaries appeared normal from day 3 of menstrual cycle for 5 days. Furthermore, from the day 7 of cycle 75 IU of hMG (Menopur, Ferring SAS, Switzerland) was injected intramuscularly for a period of at least three days depending on ovarian response. Patients were re-evaluated by TVS to assess the ovarian response on the tenth day. TVS was performed every two to three days if necessary until at least one follicle ≥ 18mm was seen. In that case, 10 000 IU of human chorionic gonadotropin (Pregnyl, 5000IU, MSD,Greek) was injected intramuscularly. However, if more than three dominant follicles were seen, the cycle would have been cancelled to prevent ovarian hyper stimulation syndrome (OHSS).

Semen preparation was performed using the standard swim up technique in the same lab. Then IUI was done once by IUI catheter (Rocket Medical, Watford, UK) attached to a 1-ml syringe, 36 hours after HCG administration. The study group (A, n=127) received luteal phase support in the form of vaginal progesterone, (400 mg twice a day), while the control group (B, n=126) received a placebo, which both started two days after the administration of hCG, and this was continued until a pregnancy test was performed. Both drugs were made by the Aburaihan Pharmaceutical Company, Tehran, Iran and were similar in appearance. The luteal phase support was continued in the two groups until the eighth week, if the pregnancy test was positive. 

The data were analyzed by SPSS software version 20 (Statistical Product and Service Solutions, SPSS Inc., Chicago), chi-square, and T test, while p≤0.05 was regarded statistically significant. Results are presented as mean ± SD and percentages.

## Results

In total, 253 women went through ovarian stimulation with clomiphene citrate (100 mg) and hMG (75 IU) for an IUI cycle. There were 127 women in the progesterone group and 126 in the placebo group. Demographic characteristics of the patients in the two groups are reported in Table 1.

Two groups were comparable regarding age, BMI, cause of infertility, and the duration of infertility.

We also compared the basal FSH and LH levels, the number of dominant follicules≥18mm and the serum progesterone on HCG day between progesterone and placebo groups. There was no statistically significant difference regarding thesevariables.

Treatment outcomes depicted in Table 2. The chemical and clinical pregnancy rate was not significantly different in group receiving vaginal progesterone comparing with placebo (15.75% vs. 12.69%, respectively; P = 0.30). The abortion rate was also the same in the progesterone and placebo group (10% vs. 18.75%, respectively; P = 0.45.)

In patients older than 30 years, the pregnancy rate after luteal phase support with progesterone was significantly higher than in the younger women aged ≤30 years (P = 0.018).

## Discussion

In this study, luteal phase support with vaginal progesterone and vaginal placebo was compared in patients undergoing ovarian stimulation with clomiphene citrate and hMG in IUI cycles.

Although the benefit of progesterone administration has been well documented in IVF (15), the question remains whether it is really necessary in mildly stimulated IUI cycles, in which 1-2 follicles have developed (17).

Erdem et al. showed that luteal phase support with progesterone increases pregnancy rates in IUI cycles stimulated with gonadotropins in patients with unexplained infertility (10).

These findings was in accordance with a similar study done by Agha-Hosseini et.al , who indicated for better pregnancy outcomes in unexplained infertility when supported with vaginal progesterone (18).

Luteal phase LH levels were found to be reduced in hMG-only cycles, which also indicate that defective LH secretion might induce a luteal phase defect in hMG stimulated cycles(9).

In another study, Maher described the cycle in which recombinant follicular stimulating hormones (r-FSH) were used for ovulation induction, and luteal phase support with vaginal progesterone improved the success of the IUI cycles (19).

On the contrary we did not find any significant difference in terms of chemical and pregnancy rates. 

Kyrou et al. concluded that luteal phase supplementation with vaginal progesterone did not improve pregnancy rates in normo-ovulatory women stimulated with clomiphene citrate for IUI, but mentioned that their study was under-powered in regards to detecting the difference in the ongoing pregnancy rate (17).

**Table 1:** Demographic characteristics of patients in both groups

**Variable**	**Progesterone group**	**Placebo group**	**p-value**
	**(n = 127)**	**(n = 126)**	
Age (yrs.) a	27.53 ± 4.23	28.11 ± 4.62	0.30
BMI (kg/m^2^) a	22.7 ± 2.8	22.6± 2.6	0.25
Duration of Infertility (yrs) a	4.55 ± 3.35	4.71 ± 3.34	0.70
Progesterone level on	0.7 ±0.3	0.6 ± 0.3	0.65
hCG day (ng/ml) a			
Basal level of FSH (mIu/ml) a	5.98 ± 2.33	6.38 ± 2.56	0.19
Basal level of LH (mIu/ml) a	8.15 ± 5.90	8.28 ± 2.96	0.82
No of Dominant follicles^a^	2.21 ± 1.29	2.43 ± 1.31	0.18
Cause n (%)^b^			
Unexplained	33 (26)	31 (25)	0.93
anovulatory	69 (54)	68 (54)	
Male	25 (20)	27 (21)	

a Values are reported as mean ± SD and were analyzed by student's test;

b Values were analyzed by χ2 test

**Table 2:** Treatment Outcomes

**Variable**	**Progesterone group**	**Placebo group**	**p-value**
	**(n = 127)**	**(n = 126)**	
Chemical Pregnancy rate^b^ n (%)	39 (30.76)	28 (22.22)	0.15
Clinical Pregnancy rate^b^ n (%)	20 (15.75%)	16 (12.69%)	0.30
Abortion rate^b^ n (%)	2 (10)	3 (18.75)	0.45
Ongoing pregnancy rate^b^ n (%)	18 (46.2)	13 (46.4)	0.98

Values were analyzed by χ2 test; p ˂ 0.05 was considered statistically significant

It is worth to emphasize that all of the above mentioned study compared supported cycles with unsupported luteal phase. While, we take into consideration the placebo effect by using placebo in control group, which is the strength of our study. 

On the other hand, it was shown that LH secretions are reduced to almost undetectable levels shortly after the ovulatory hCG injection, and remain low for the whole length of the luteal phase, which indicates that defective LH secretion might induce a luteal phase defect in stimulated cycles (10), so it was reasonable for us using progesterone in our study, for luteal phase support irrespective of the type of stimulatory drug (clomiphene or hMG or both).

However, Montville et al. came to the conclusion that luteal phase support with progesterone in women suffering from PCOS stimulated with letrozole improved clinical pregnancy rates, although the results were on the contrary for women stimulated with clomiphene citrate (20).

Ragni et al. compared the luteal phase profile in patients stimulated with gonadotropin and GnRH antagonist for IUI cycles, with those without the GnRH antagonist. They demonstrated that the progesterone level was similar in the luteal phase in both groups. Considering this fact, they suggested that the GnRH antagonist is a safe procedure in gonadotropin stimulated IUI cycles without luteal phase supplementation (21). But, they used gonadotropin for induction of ovulation and not clomiphene citrate followed by gonadotropin as we used in our study.

A systematic review and meta-analysis in 2013 was performed by Hill MJ et.al which included five trials comprised 1298 patients. They concluded that luteal phase support may be of benefit to patients undergoing ovulation induction with gonadotropins but not clomiphene citrate in IUI cycles, suggesting a potential difference in endogenous luteal phase function depending on the method of ovulation induction (16).

The finding of this systematic review supported the results of Tavaniotou et al study (9).

Comparing the results of progesterone administration in women under the age 30, to women more than 30, showed the trend toward higher pregnancy rates which supported the previous hypothesis and studies (7, 22).

With regard to the previous studies about this subject the design of our study was different in three important points. First, it was comprised of all kinds of sub fertile patients who were suitable for IUI cycle. The second difference was the use of placebo in the control group. And at last, ovarian stimulation was performed by using clomiphene and gonadotropin together.

## Conclusion

Luteal phase support may be more beneficial in older women in IUI cycles. Moreover, regarding to our study design, using clomiphene in association with gonadotropin for induction ovulation in patients who undergoing IUI cycle, may result better in terms of luteal phase function and pregnancy outcomes. 

## Confilict of Interests

Authors declare that they have no conflict of interest.
